# Vaccination to Promote Healthy Aging: The Five Ws

**DOI:** 10.1093/geroni/igaa057.2926

**Published:** 2020-12-16

**Authors:** Leonard Friedland, Leonard Friedland

## Abstract

This symposium addresses the role of vaccination to promote healthy aging and the process of developing and maintaining the functional ability that enables wellbeing in older age. Adults age 65 and over are at increased risk of certain infectious diseases due to immunosenescence. Therefore, immunization of older adults against targeted infectious diseases, including pertussis, shingles, influenza, and pneumococcal disease, can help to reduce morbidity and premature mortality. Vaccines in development to protect against additional infectious diseases causing significant morbidity and mortality in older adults, such as respiratory syncytial virus, can further promote healthy aging. The population of older adults in the US is projected to grow significantly over the next 30 years, with a corresponding increase in the incidence and economic costs of vaccine-preventable diseases. Immunization of older adults is a proven, cost-effective strategy that is critical for reducing the public health impact and societal costs in an aging US population. Implementation of evidence-based recommended vaccines for older adults presents challenges, including financial barriers, addressing disparities and inequities in health care delivery for older adults, and overcoming vaccine hesitancy. We plan to review these topics and present data we have generated to support the value of vaccination in adults age 65 and over. Health Behavior Change Interest Group Sponsored Symposium.

